# Antarctic Porifera database from the Spanish benthic expeditions

**DOI:** 10.3897/zookeys.401.5522

**Published:** 2014-04-14

**Authors:** Pilar Rios, Javier Cristobo

**Affiliations:** 1 Instituto Español de Oceanografia (Centro Oceanográfico de Gijón), Avda. Principe de Asturias 70bis, 33212, Gijon, Spain

**Keywords:** Porifera, Antarctic, Deep-Sea, Biodiversity, BENTART, Antarctic Peninsula, Bellingshausen Sea, Sponges, Benthic fauna

## Abstract

The information about the sponges in this dataset is derived from the samples collected during five Spanish Antarctic expeditions: Bentart 94, Bentart 95, Gebrap 96, Ciemar 99/00 and Bentart 2003. Samples were collected in the Antarctic Peninsula and Bellingshausen Sea at depths ranging from 4 to 2044 m using various sampling gears.

The Antarctic Porifera database from the Spanish benthic expeditions is unique as it provides information for an under-explored region of the Southern Ocean (Bellingshausen Sea). It fills an information gap on Antarctic deep-sea sponges, for which there were previously very few data.

This phylum is an important part of the Antarctic biota and plays a key role in the structure of the Antarctic marine benthic community due to its considerable diversity and predominance in different areas. It is often a dominant component of Southern Ocean benthic communities.

The quality of the data was controlled very thoroughly with GPS systems onboard the R/V Hesperides and by checking the data against the World Porifera Database (which is part of the World Register of Marine Species, WoRMS). The data are therefore fit for completing checklists, inclusion in biodiversity pattern analysis and niche modelling. The authors can be contacted if any additional information is needed before carrying out detailed biodiversity or biogeographic studies.

The dataset currently contains 767 occurrence data items that have been checked for systematic reliability. This database is not yet complete and the collection is growing. Specimens are stored in the author’s collection at the Spanish Institute of Oceanography (IEO) in the city of Gijón (Spain). The data are available in GBIF.

## General description

The main objective of these surveys, within the Bentart, Gebrap and Ciemar projects, was to study benthic ecosystem biodiversity in the Bellingshausen Sea and Antarctic Peninsula, an area for which there is little information. Samples were taken for quantification of infauna and epibenthos paying particular attention to suprabenthos, meiofauna and demersal fish.

The preliminary data analysis showed that there was very little epibenthic and suprabenthic fauna in the Bellingshausen Sea compared to the northern areas (Antarctic Peninsula and South Shetland Islands).

As there are few collections available in the GBIF database the present collection improves scientific knowledge of the Porifera in the Antarctic Peninsula and Bellingshausen Sea and provides new and important information on the distributions of the species.

This phylum is an important part of the Antarctic biota and plays a key role in the structure of the Antarctic marine benthic community due to its considerable diversity and predominance in different areas. It is often a dominant component of Southern Ocean benthic communities.

The quality of the data was controlled very thoroughly and therefore the data are fit for completing checklists, inclusion in biodiversity pattern analysis and niche modelling. The database fills an information gap on deep-sea sponges from the Southern Ocean, for which there were previously very few data.

The dataset currently contains 767 occurrence data items that have been checked for systematic reliability. This database is not yet complete and the collection is growing. Specimens are stored in the author’s collection at the Spanish Institute of Oceanography (IEO) in the city of Gijón (Spain). The data are available in GBIF.

## Project details

**Project title:** Benthic biodiversity of the Bellingshausen Sea and Antarctic Peninsula: Porifera

**Personnel:** Pilar Rios and Javier Cristobo

**Funding:** This database has been supported through the following projects:

1. Estudios de la fauna y flora bentónica de los fondos de la zona sur de la isla Livingston (Shetlands del Sur), Antártida. (Project ANT93-0996).
2. Estudios de la fauna y flora bentónica de los fondos de la zona sur de la isla Livingston y áreas adyacentes (Shetlands del Sur, Antártida. (Project ANT94-1161-E).
3. Fauna y Flora Bentónicas de los fondos del Sur de la Isla Livingston y áreas adyacentes: estudio del material biológico y datos recogidos en las campañas nacionales del Bentos Antártico. (Project ANT95-1011).
4. Muestreos bentónicos en áreas volcánicas de la cuenca del Bransfield (Antártida). (Project ANT96-2440-E).
5. Ampliación del Proyecto: Fauna y flora bentónica de los fondos de la zona sur de isla Livingston y áreas adyacentes. Estudio del material biológico y datos recogidos en las dos campañas españolas de bentos antártico (Project ANT97-2097-E).

Estudio integrado de la biodiversidad bentónica del Mar de Bellingshausen y Península Antártica (Antártida del Oeste) (Segunda Campaña de Muestreo a bordo del BIO Hespérides) Project MCYT REN2003-01881/ANT.

**Study area:** The study area was the western sector of the Antarctic (Bellingshausen Sea, Peter I Island and Antarctic Peninsula), in particular the continental shelf, upper slope and deep-sea basins of the Bellingshausen Sea. There is very little information on this sector and the sampling showed that there are interesting sponge species here. According to a recent gap analysis carried out by Griffiths et al. (2010) the deep-sea zone of the Southern Ocean is in general a very under sampled area.

**Design:** The Antarctic sector corresponding to the Bellingshausen Sea is one of the most difficult areas to access with a research vessel due to the prevalence of ice most of the year (Clarke and Johnston 2003). Peter I Island (68°S) is an isolated oceanic island that lies 450 km north of Eights Coast at about 4 km water depth. The island has a maximum length of 20 km and rises to a height of 1 640 m. It is composed of different volcanic rocks although most of its surface is glaciated.

The BENTART Projects were carried out from 1994 to 2006 in four oceanographic surveys onboard the R/V Hespérides and provided valuable information on the Antarctic benthic biodiversity in the South Shetlands, Antarctic Peninsula and Bellingshausen Sea. The methodological approach used was totally innovative and consisted in sampling transects that were perpendicular to the coast at stations with a previously established bathymetric layer. The large-scale variations in the different biodiversity and abundance indices were established along latitudinal and bathymetric gradients. Quantitative multiple sampling of the three benthic compartments (endofauna, epibenthos and suprabenthos) using specific sampling gears (box corer, Agassiz trawl and suprabenthic sled) in the water column and sediment at the same stations made it possible to analyse both the biotic and abiotic data as well as gain an overall view of the benthic ecosystems and factors that regulate their distribution.

During the GEBRAP 96 survey nine trawls at 647 to 1592 m in depth were carried out on underwater volcanic structures located in the central basin of Bransfield Strait. The objective was to collect lithological samples and associated benthic fauna exposed to hydrothermalism.

The macrobenthos from the Bransfield and Gerlache Straits was sampled in the CIEMAR 99/00 expedition onboard the R/V Hespérides. Invertebrate fauna was collected at 14 stations using a rock dredge with 80 cm and 30 cm horizontal and vertical openings and a 10 mm mesh size.

Porifera are one of the most important elements in the Antarctic biota due to their considerable diversity and predominance in different areas (Sarà et al. 1992). In the Antarctic benthos at depths of 100 m these sponges can attain a biomass comparable to the largest sponges from tropical areas (Beliaev and Ushakov 1957). Their large size and that of their spicules, as well as their uniform distribution, are the main aspects that characterize them. These areas are rich in silica sponges (Demospongiae and Hexactinellida) and there are very few calcareous sponges. In this project we computerized and georeferenced the database of specimens collected during the Spanish expeditions Bentart 94, Bentart 95, Bentart 03, Gebrap 96 and Ciemar 99/00.

**Data published at GBIF:** http://www.gbif.es:8080/ipt/resource.do?r=poriferabentart

## Taxonomic coverage

This database is devoted to the Porifera collected in the Spanish Antarctic Expeditions (Bentart, Gebrap and Ciemar) in the Antarctic Peninsula and Bellingshausen Sea onboard the R/V Hesperides. The Antarctic sponge species are characterized by an overall endemism of 43% with a higher level for the class Hexactinellida (68%) and a few endemic genera dominating shelf communities, most notably in the Weddell and Ross Seas. The Demospongiae class has the highest number of species in the Antarctic Porifera and has been recorded in all regions of the Antarctic. Calcareous sponges are the least abundant and least studied class of sponges in the Antarctic.

**General taxonomic coverage:** The sponges of the Antarctic and neighbouring oceanographic regions were assessed for species richness and biogeographic patterns based on over 8800 distribution records (Downey et al. 2012). Sampling intensity has varied greatly in the Antarctic. Sampling hotspots are the Antarctic Peninsula, South Georgia, north New Zealand and Tierra del Fuego. There has been little sampling carried out in the Bellingshausen and Amundsen Seas in the Southern Ocean.

Previous estimates of the number of sponge species in the Southern Ocean (SO) vary between 250 and 530. All four classes of Porifera (Hexactinellida, Demospongiae, Homoscleromorpha and Calcarea) are represented in the SO. There is a higher diversity and abundance of the first two classes (particularly the demosponges) compared to the last two. Recent studies (Downey et al. 2012) have found that there are 397 sponge species from the Southern Ocean, representing 139 genera in 70 families.

This dataset focuses on Antarctic Porifera and includes data on nine orders. The highest level of identification is Order Poecilosclerida with 53 species (577 samples). We identified five species of Lyssacinosida (123 samples), one species of Leucosolenida (2 samples), one species of Chondrosida (1 sample), three species of Spirophorida (70 samples), six species of Hadromerida (138 samples), two genera of Halichondrida (64 samples), five species of Haplosclerida (157 samples), one species of Dendroceratida (19 samples) and 63 records identified at class level.

## Taxonomic ranks

**Kingdom:** Animalia

**Phylum:** Porifera

**Class:** Hexactinellida, Demospongiae and Calcarea

**Order:** Chondrosida, Dendroceratida, Hadromerida, Halichondrida, Haplosclerida, Leucosolenida, Lyssacinosida, Poecilosclerida, Spirophorida

**Family:** Acarnidae, Axinellidae, Chalinidae, Cladorhizidae, Clathriidae, Coelosphaeiridae, Darwinnellidae, Dendoricellidae, Desmacellidae, Grantiidae, Guitarridae, Halichondriidae, Halisarcidae, Hymedesmiidae, Iotrochotidae, Isodictyidae, Latrunculidae, Microcionidae, Mycalidae, Myxillidae, Niphatidae, Petrosiidae, Phloeodictyidae, Polymastiidae, Raspailidae, Rossellidae, Stylocordylidae, Suberitidae, Tedaniidae, Tetillidae, Timeidae

**Common names:** Sponges

## Spatial coverage

### General spatial coverage

**Figure 1.**
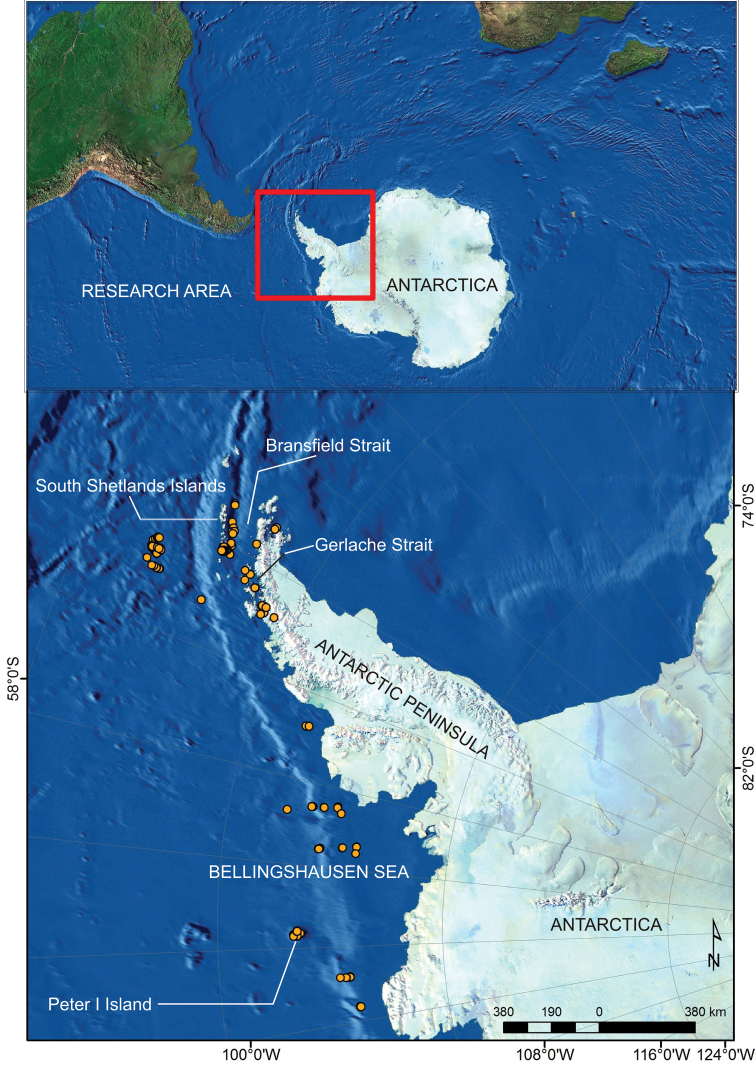
Bentart, Gebrap and Ciemar stations in the Bellingshausen Sea, Peter I Island and Antarctic Peninsula (South Shetland Islands, Bransfield and Gerlache Straits).

The sampling area ranged from 70°53'S to 60°19'S latitude and from 57°01"W to 98°28"W longitude (Figure 1).

**Figure 2.**
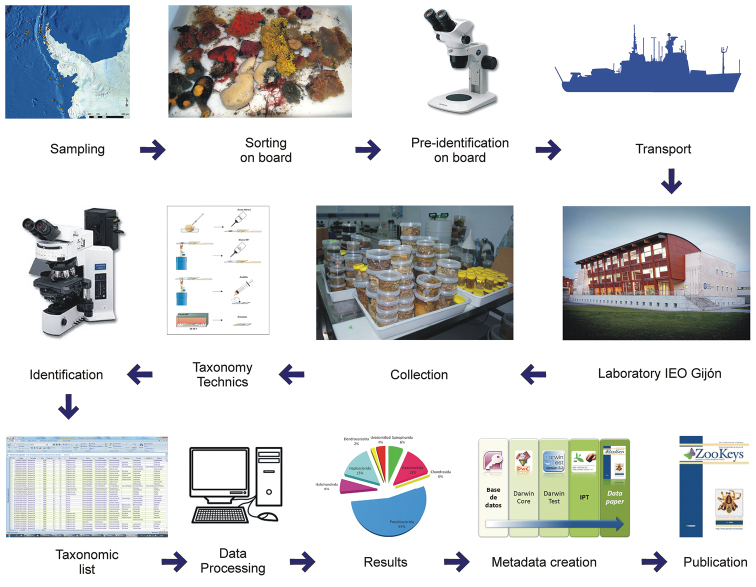
Summary of the procedure used to generate the dataset.

**Figure 3.**
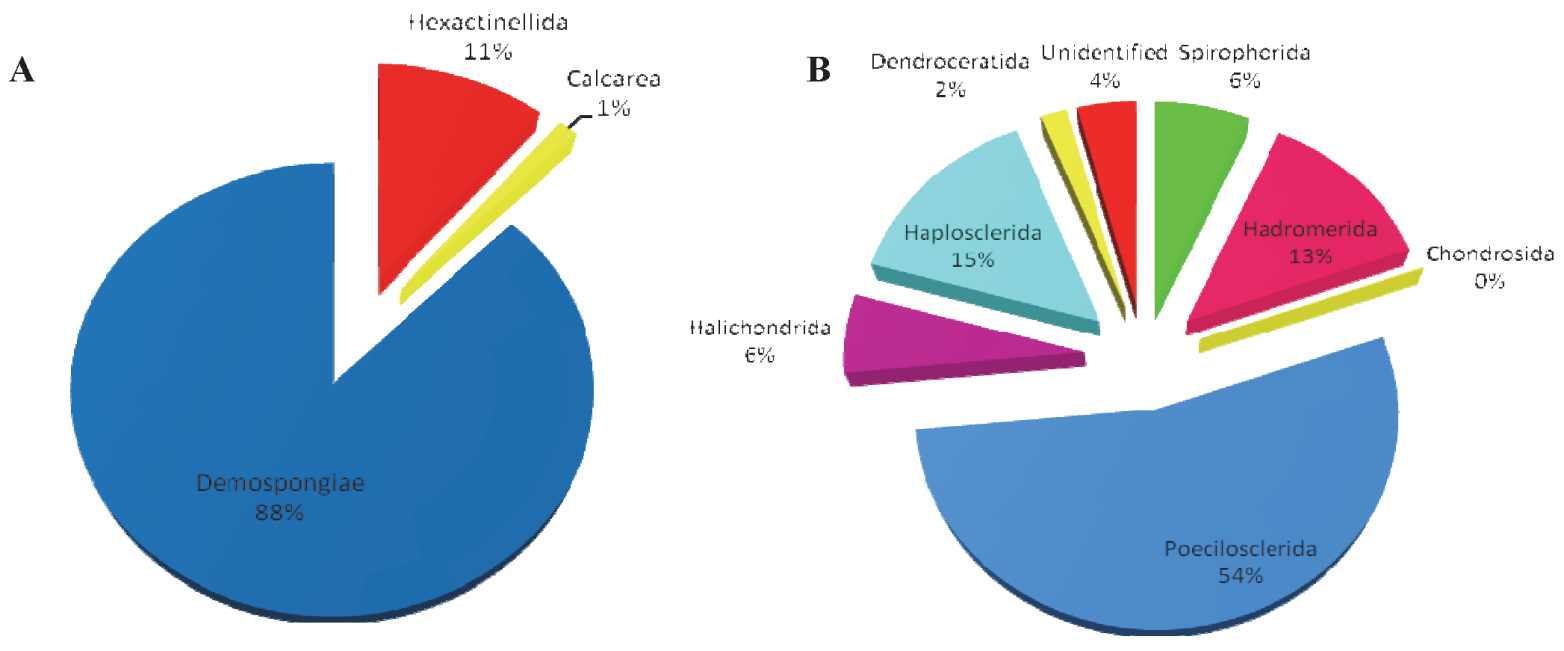
**A** Distribution range of the Porifera classes **B** Orders within the class Demospongiae.

### Coordinates

70°53'31"S and 60°19'08”S latitude; 57°01'10"W and 98°28'54"W longitude.

### Temporal coverage:

Bentart expeditions: 1994–2003

Gebrap expedition: 1996

Ciemar expedition 1999–2000

## Description of the natural collections

**Parent collection identifier:** Instituto Español de Oceanografia (IEO) Gijon Porifera

**Collection name:** IEO Gijon Porifera BENTART

**Collection identifier:** Pilar Ríos, Javier Cristobo. http://www.gbif.es:8080/ipt/

**Specimen preservation method:** Ethanol

## Methods

**Steps:**

– The material examined onboard the R/V Hesperides was collected with different methods: rock dredge, Van Veen dredge, anchor dredge, suprabenthic sledge, fish traps, scuba diving, box corer and Agassiz trawl.
– Once on deck, the contents of the samplers were immediately sorted by taxa, washed in cold sea water and classified by a specialist in each group. Sponge samples were then placed in 80% ethanol.
– The taxonomic identification was performed in the laboratory using an optical microscope and a Scanning Electron Microscope when necessary.

**Study area:** The Southern Ocean with particular emphasis on the coastal shelf areas of the Bellingshausen Sea and Antarctic Peninsula as well as the Peter I, Deception and Livingston islands, without specific temporal sampling patterns.

**Sampling:** Porifera were collected during oceanographic cruises in the Antarctic Ocean from 1994 to 2003 as part of the Spanish expeditions Bentart 94, Bentart 95, Bentart 03, Gebrap 96 and Ciemar 99/00. The database has been upgraded with data collected in 2013. Specimens were fixed in 4% formaldehyde or 70% ethanol and then preserved in 70% ethanol. Treatment in formalin made it possible to carry out cytological studies. In the laboratory the organic matter was digested with nitric acid taken to boiling point according to the methods developed by Rützler (1978) and Cristobo et al. (1993). The spicules of some samples were examined using a Leica S440 Scanning Electron Microscope.

**Quality control:** Systematic reliability and consistency were checked by Pilar Rios and Javier Cristobo. Identification was based on species descriptions by different authors compiled in Systema Porifera (Hooper and Soest 2002). The doctoral thesis by Rios (2007) was used to check Antarctic species. The classification system proposed by Van Soest and Hajdu (2002) was used.

## Datasets

### Dataset description

**Object name:** Darwin Core Archive Antarctic Porifera database from the Spanish benthic expeditions

**Character encoding:** UTF-8

**Format name:** Darwin Core Archive format

**Format version:** 1.0

**Distribution:** http://www.gbif.es:8080/ipt/archive.do?r=poriferabentart

**Publication date of data:** 2013-05-14

**Language:** Spanish

**Licenses of use:** This Antarctic Porifera database from the Spanish benthic expeditions is made available under the Open Database License: http://opendatacommons.org/licenses/odbl/1.0/. Any rights in individual contents of the database are licensed under the Database Contents License: http://opendatacommons.org/licenses/dbcl/1.0/

**Metadata language:** English

**Date of metadata creation:** 2013-03-20

**Hierarchy level:** Dataset

## References

[B2] Ríos P Cristobo FJ Urgorri V (2004) Poecilosclerida (Porifera, Demospongiae) collected by the Spanish Antarctic expedition BENTART-94. Cahiers de Biologie Marine 45: 97-11

[B3] Ríos P Cristobo FJ (2006) A new species of *Biemna* (Porifera: Poecilosclerida) from Antarctica: *Biemna strongylota*. Journal Marine Biological Association UK 86: 949-955. doi: 10.1017/S0025315406013919

[B4] Ríos P Cristobo FJ (2007) A new species of *Phorbas* (Porifera: Poecilosclerida) from the Bellingshausen Sea, Antarctica. Journal Marine Biological Association U.K. 87: 1485-1490. doi: 10.1017/S0025315407058079

[B5] Ríos P Cristobo FJ (2007) Sponges of genus *Myxilla* Schmidt, 1862, collected in Antarctic waters by Spanish Antarctic expeditions. Porifera Research: Biodiversity, Innovation and Sustainability 1: 525-546

[B6] Ríos P (2007) Esponjas del Orden Poecilosclerida de las campañas españolas de bentos antártico. Tesis Doctoral Universidad de Santiago de Compostela, 527 pp

[B8] Beliaev GM Ushakov PV (1957) Certain regularities in the quantitative distribution of the bottom fauna in Antarctic waters. American Institute of Biological Sciences 112: 116-119

[B9] Clarke A Johnston NM (2003) Antarctic Marine Benthic diversity. Oceanography and Marine Biology: an Annual Review 41: 47-114

[B10] Cristobo FJ Urgorri V Solórzano MR Ríos P (1993) Métodos de recogida, estudio y conservación de las colecciones de poríferos. In: Palacios F Martínez C Thomas B (Eds) International Symposium and First World Congress on Preservation and Conservation of Natural History Collections, Madrid 2 Dirección General de Bellas Artes y Archivos Ministerio de Cultura, Madrid, 277-287

[B11] Downey RV Griffiths HJ Linse K Janussen D (2012) Diversity and Distribution Patterns in High Southern Latitude Sponges. PLoS ONE 7(7): . doi: 10.1371/journal.pone.0041672 10.1371/journal.pone.0041672 PMC3404021 22911840

[B12] Griffiths HJ Danis B Clarke A (2010) Quantifying Antarctic marine biodiversity: The SCAR-MarBIN data portal. Deep Sea Research Part II: Topical Studies in Oceanography (October): 1–12

[B13] Hooper J Soest Rv (Eds) (2002) Systema Porifera: A Guide to the Classification of Sponges. Kluwer Academic/Plenum Publishers, New York, 2002.

[B14] Ríos P Cristobo J (2011) La Exploración Antártica In: La Antártida, la vida en el límite, las exploraciones Bentart. Hercules de Ediciones ISBN: 978-84-92715-29-9: 50–75

[B15] Ríos P Cristobo FJ (2011) Poriferos In: La Antártida, la vida en el límite, las exploraciones Bentart. Hercules de Ediciones ISBN: 978-84-92715-29-9: 256

[B16] Rützler K (1978) Sponges on coral reefs. In: Stoddart DR Johanness RE (Eds) Coral reefs: research methods. Unesco, Paris, 81–120

[B17] Sarà M Balduzzi A Barbieri M Bavestrello G Burlando B (1992) Biogeographic traits and checklist of Antarctic demosponges. Polar Biology 12: 559-585. doi: 10.1007/BF00236980

[B18] Van Soest RWM Hajdu E (2002) Systema Porifera: A Guide to the Classification of Sponges, New York, 1810 pp

